# Cannabis Use Disorder and Risk of Pancreatic Cancer in Patients with Chronic Pancreatitis: a Multicenter Retrospective Cohort Study

**DOI:** 10.1007/s12029-025-01383-w

**Published:** 2026-01-14

**Authors:** Muhammad Hassaan Arif Maan, Soban Maan, Muhammad Mursaleen Ahmad, Ritik Mahaveer Goyal, Sunnia Khan, Muhammad Waleed, Imran Qureshi, Kaveh Hajifathalian, Ahmed Al-Khazraji

**Affiliations:** 1 Department of Medicine, Rutgers New Jersey Medical School, Newark, NJ 07103 USA; 2 Department of Medicine, Division of Gastroenterology & Hepatology, West Virginia University, Morgantown, West Virginia USA; 3 Pakistan Institute of Medical Sciences, Islamabad, Pakistan; 4 Punjab Medical College, Faisalabad, Pakistan; 5 Department of Medicine, Division of Gastroenterology & Hepatology, Rutgers New Jersey Medical School, New Jersey, Newark USA

**Keywords:** Cannabis use disorder, Chronic pancreatitis, Pancreatic cancer, Acute pancreatitis, Cannabinoids

## Abstract

**Background:**

Cannabis use is increasing globally, with a parallel rise in Cannabis Use Disorder (CUD). Chronic pancreatitis (CP), a progressive inflammatory condition, is associated with acute pancreatitis (AP) flares and an elevated risk of pancreatic cancer (PC). Although cannabis is often used for pain management in CP, its impact on PC risk and AP flare frequency is unclear.

**Methods:**

We conducted a retrospective cohort study using TriNetX to identify adults with CP, stratified by CUD status. Patients with pre-existing PC were excluded. Propensity score matching (1:1) was applied for demographics, behavioral factors, and comorbidities. The primary outcome was PC incidence; the secondary was AP flare frequency. Hazard ratios (HR) were calculated using Cox proportional hazards regression. Sensitivity analysis adjusted for opioid use disorder.

**Results:**

Before matching, the CUD cohort (*n* = 10,864) had higher rates of alcohol and nicotine use than controls (*n* = 42,160). After matching, 6,858 patients per group remained with balanced covariates (SMD < 0.1). Mean follow-up was shorter in the CUD cohort (736 ± 422 vs. 896 ± 368 days). CUD was associated with a significantly reduced risk of PC (67 vs. 274 cases; HR: 0.263, 95% CI: 0.202–0.344; *p* < 0.001) but a modest increase in AP flare risk (HR: 1.102, 95% CI: 1.043–1.166; *p* = 0.001). Results were consistent in the sensitivity analysis.

**Conclusions:**

Among patients with CP, CUD was associated with lower rates of PC detection during available follow-up, but a slightly increased risk of AP flares. These findings warrant further prospective and mechanistic studies to clarify cannabis’s role in pancreatic disease.

## Introduction

Cannabis use is reported in about 2.5% of the world’s population, and about 45% of individuals in the US report lifetime cannabis use. The prevalence is gradually rising with the widespread legalization of its medical and recreational use and is associated with a simultaneous increase in cannabis use disorder (CUD) [1]. This growing prevalence underscores the importance of understanding the potential health effects of cannabis use, particularly in individuals with preexisting conditions. The Diagnostic and Statistical Manual of Mental Disorders, Fifth Edition (DSM-5) characterizes cannabis use disorder by a problematic cannabis use pattern leading to clinically significant impairment or distress. This requires meeting 2 out of 11 criteria within a 1-year period, including social impairment, risky use, impaired control over use, tolerance, and withdrawal [2, 3]. 

Chronic pancreatitis (CP) is progressive pancreatic inflammation leading to irreversible damage and persistent abdominal pain. CP is associated with an elevated risk of pancreatic cancer (PC), a malignancy with one of the highest mortality rates among cancers [4]. 

CP patients may use cannabis as an alternative or adjunct therapy for pain management. While clinical efficacy data in CP specifically remain limited, medical cannabis has been studied for chronic pain conditions more broadly [5–7]. Despite the known association between CP and PC, a paucity of data explores the relationship between CUD and PC in individuals with CP. This study aims to address this by conducting a retrospective cohort study.

## Methods

We conducted a retrospective cohort study utilizing TriNetX, a research network that provides real-time access to electronic health records from participating healthcare organizations. Most of these organizations are in the United States. Since it only provides de-identified data, TriNetX is compliant with the Health Insurance Portability and Accountability Act (HIPAA) and has received a waiver of review and informed consent from the Western Institutional Review Board. We identified patients using International Classification of Diseases, Tenth Revision (ICD-10) diagnostic codes available within the TriNetX research network.

### Study Design and Participants

Patients aged 18 years or older with a CP, defined by ICD-10 codes K86.0 and K86.1, were included in the study. Patients were stratified into two cohorts: those with CUD, identified using ICD-10 codes F12.1 and F12.2, and a control cohort without cannabis-related disorders (F12). Patients with pre-existing PC (ICD-10 C25) were excluded. Follow-up was completed through December 2024.

### Study Definitions and Outcomes

The primary outcome was the development of PC. The secondary outcome was the occurrence of AP flares, identified using ICD-10 codes K85 and K85.92.

### Statistical Analysis

We used TriNetX’s in-built capabilities to conduct our analyses. To control for confounding, we performed 1:1 propensity score matching for demographic factors (age, sex, race, ethnicity), behavioral factors (alcohol use, nicotine dependence), and comorbidities (cholelithiasis, obesity, type 2 diabetes, hypertension, hyperlipidemia, chronic kidney disease, and systemic connective tissue disorders). Covariate balance between the two groups was evaluated using standardized mean difference (SMD), with a value < 0.1 indicating adequate balance.

After matching, Cox proportional hazards model (a time-to-event survival analysis which inherently accounts for differences in follow-up time by estimating the relative risk of the event occurring at any given point during the follow-up period) was used to calculate hazard ratios (HR) and 95% confidence intervals (CIs) (R’s Survival package v3.2-3; R Foundation for Statistical Computing). Kaplan-Meier analysis was also conducted with log-rank tests to compare outcomes between the cohorts.

### Sensitivity Analysis

Opioid use disorder (OUD), identified by ICD-10 F11, is a common CUD-associated comorbidity that can potentially impact outcomes. Hence, to test the robustness of our findings, we conducted a sensitivity analysis after including opioid use disorder in the covariates used for propensity matching.

## Results

The CUD cohort had 10,864 patients and the control cohort had 42,160 patients. The CUD cohort was a younger population (46.4 ± 13.0 years vs. 53.3 ± 18.1 years, SMD: 0.045) and had a higher proportion of females (50.9% vs. 34.9%, SMD: 0.501) and African American individuals (30.6% vs. 12.1%, SMD: 0.538). On the other hand, the proportion of white (52.9% vs. 64.8%, SMD: 0.324) and Asian individuals (1.0% vs. 3.5%, SMD: 0.114) was significantly lower in the CUD cohort. Prevalence of comorbid conditions such as cholelithiasis (12.1% vs. 7.7%, SMD: 0.393), type 2 diabetes (30.7% vs. 17.8%, SMD: 0.619), hypertension (56.8% vs. 28.9%, SMD: 0.883), hyperlipidemia (26.7% vs. 15.2%, SMD: 0.478), alcohol-related disorders (53.7% vs. 8.0%, SMD: 1.153), and nicotine dependence (64.9% vs. 9.0%, SMD: 1.337) were also significantly higher in the CUD cohort. Similarly, chronic kidney disease (13.8% vs. 6.7%, SMD: 0.422) and overweight/obesity (18.3% vs. 6.3%, SMD: 0.399) were significantly more prevalent in the CUD cohort. (Table 1)

**Table 1:** Cohort characteristics before and after propensity score matching

Characteristic	Before matching: CUD cohort (*n* = 10,894)	Before matching: control cohort (*n* = 42,160)	*P*-value (Before)	Std. Diff. (Before)	After matching: CUD cohort (*n* = 6,858)	After matching: control cohort (*n* = 6,858)	*P*-value (After)	Std. Diff. (After)
Demographics								
Age at Index (years)	46.4 ± 13.0	53.3 ± 18.1	< 0.001	0.442	47.0 ± 13.4	48.1 ± 14.6	< 0.001	0.081
Female (%)	34.9%	50.9%	< 0.001	0.328	38.6%	37.0%	0.055	0.033
White (%)	52.9%	64.8%	< 0.001	0.243	56.9%	55.8%	0.185	0.023
American Indian or Alaska Native (%)	0.6%	0.2%	< 0.001	0.055	0.5%	0.5%	0.903	0.002
Native Hawaiian or Other Pacific Islander (%)	0.6%	0.3%	< 0.001	0.042	0.5%	0.6%	0.728	0.006
Black or African American (%)	30.6%	12.1%	< 0.001	0.463	24.5%	26.8%	0.002	0.053
Hispanic or Latino (%)	6.4%	5.6%	0.001	0.035	6.6%	5.9%	0.097	0.028
Asian (%)	1.0%	3.5%	< 0.001	0.172	1.2%	0.9%	0.046	0.034
Diagnoses								
Overweight and Obesity (%)	18.3%	6.3%	< 0.001	0.370	14.4%	14.9%	0.385	0.015
Type 2 Diabetes Mellitus (%)	30.7%	17.8%	< 0.001	0.302	28.2%	29.5%	0.093	0.029
Essential Hypertension (%)	56.8%	28.9%	< 0.001	0.590	48.5%	51.5%	< 0.001	0.061
Alcohol-Related Disorders (%)	53.7%	8.0%	< 0.001	1.137	36.4%	36.0%	0.606	0.009
Nicotine Dependence (%)	64.9%	9.0%	< 0.001	1.420	45.9%	45.7%	0.824	0.004
Chronic Kidney Disease (%)	13.8%	6.7%	< 0.001	0.235	11.9%	12.2%	0.564	0.010
Hyperlipidemia, Unspecified (%)	26.7%	15.2%	< 0.001	0.286	23.8%	25.1%	0.077	0.030
Cholelithiasis (%)	12.1%	7.7%	< 0.001	0.148	10.4%	11.0%	0.270	0.019
Systemic Connective Tissue Disorders (%)	2.8%	2.1%	< 0.001	0.041	2.8%	2.9%	0.538	0.011

**Fig. 1:**
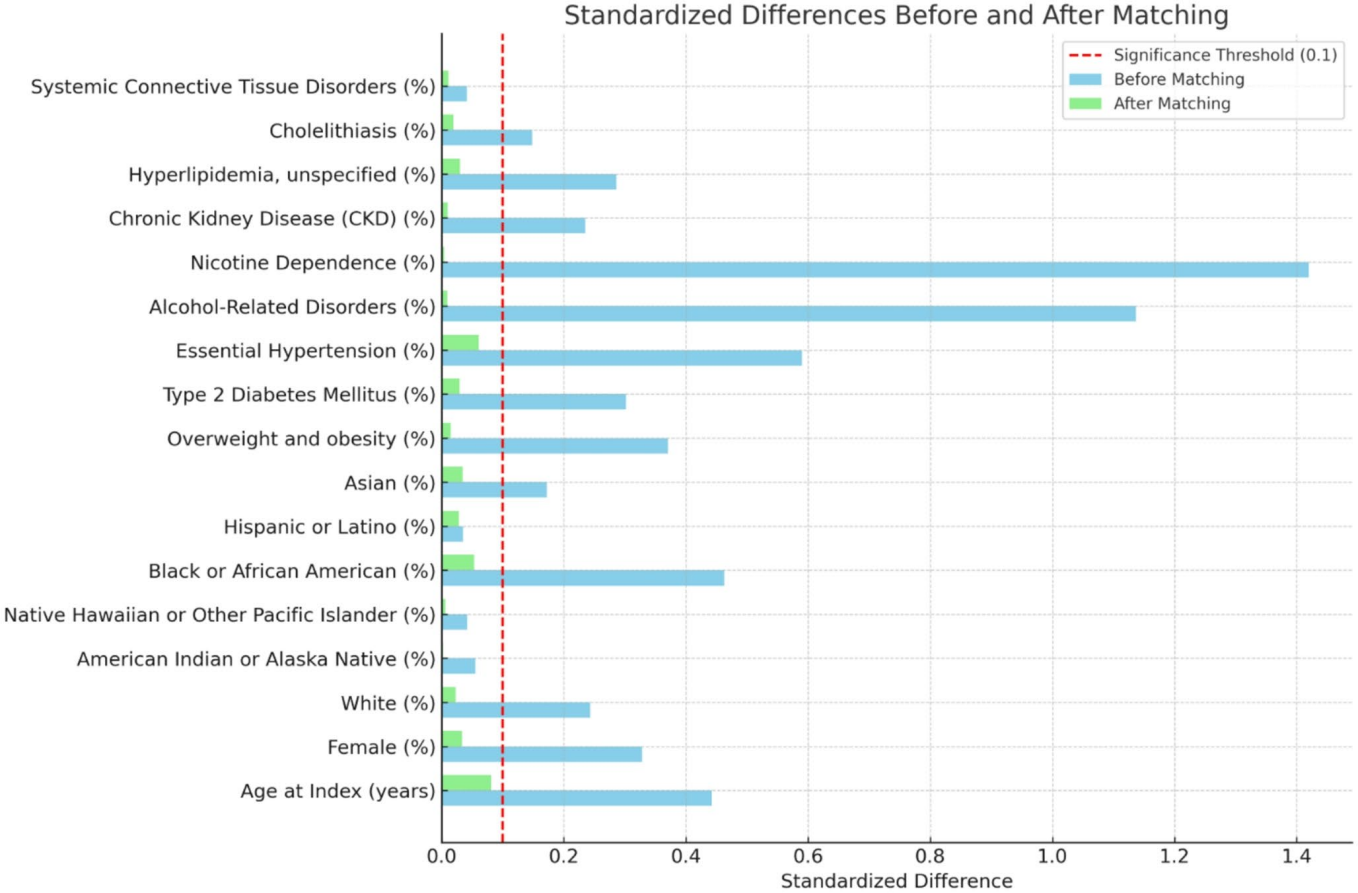
Effect of propensity score matching on baseline cohort characteristics

Following matching, both cohorts included 6,858 patients, with appropriate balancing of all covariates (SMD < 0.1). (Fig. 1) The mean follow-up was 736 ± 422 days for the CUD cohort and 896 ± 368 days for the control cohort. Given the non-normal distribution of follow-up duration, medians are also reported: 642 days (IQR 381–1,012) in the CUD cohort vs. 811 days (IQR 501–1,219) in the control cohort.

## Outcomes

The CUD cohort showed significantly lower rates of PC detection (67 vs. 274; HR 0.263, 95% CI: 0.202–0.344, log-rank p-value: <0.001). (Table 2) Conversely, the risk of AP flares was higher in the CUD cohort vs. controls (2,487 vs. 2,446; HR 1.102, 95% CI: 1.043–1.166, log-rank p-value: 0.001). In sensitivity analysis adjusting for opioid use disorder, results remained consistent with the primary analysis (PC: 62 vs. 328 h 0.281, 95% CI: 0.213–0.372, *p* < 0.001), demonstrating robustness of our findings.

**Table 2:** Comparison of outcomes between CUD cohort and control cohort

Outcome	Patients with outcome in CUD cohort (*n*)	Patients with outcome in control cohort (*n*)	Hazard ratio (95% CI)	Log rank *p*-value
Pancreatic Cancer	67	274	0.263 (0.202–0.344)	< 0.001
Acute Pancreatitis Flare	2,487	2,446	1.102 (1.043–1.166)	0.001

## Discussion

The relationship between CUD and cancer risk remains complex and not fully established. With the growing prevalence of cannabis use, understanding its health implications is increasingly critical. Cannabis use has been associated with both an increased risk of some cancers, such as particular testicular cancers, and a decreased risk of others, such as bladder cancer [8, 9]. This variation in outcomes is often influenced by the type of cannabinoid being used and the mode of consumption. For instance, smoking cannabis is believed to increase the risk of cancers in the throat and respiratory tract due to the presence of carcinogens in cannabis smoke, which resemble those found in cigarette smoke [8, 10]. 

The relationship between cannabis use and cancer risk remains incompletely understood. Preclinical studies demonstrate that certain cannabinoids can inhibit tumor growth, induce apoptosis, and reduce angiogenesis in pancreatic cancer cell lines and animal models. However, several important caveats limit the interpretation of these findings. Most anti-cancer studies have been conducted in vitro or in animal xenograft models rather than clinical trials [11–14]. Cannabinoid receptors (CB1 and CB2) are expressed ubiquitously throughout the body and bind endocannabinoids, phytocannabinoids (such as THC and CBD), and synthetic cannabinoids—each with potentially different biological effects [15]. Studies demonstrating synergistic effects with chemotherapy have predominantly used synthetic cannabinoids rather than the cannabis products consumed by patients with CUD [16]. The cannabis used by patients in our cohort likely differs substantially from purified compounds studied in laboratory settings in terms of cannabinoid composition, THC: CBD ratios, route of administration, and dose.

We also found a modestly increased risk of AP flares in CP in the CUD. Whereas growing evidence suggests that cannabis use may contribute to idiopathic acute pancreatitis (AP), our study is unique in demonstrating an increased risk of AP flares, specifically within the CP population [17]. This is a pertinent novelty, as CP patients are inherently at higher risk for recurrent pancreatic inflammation, with a pressing need for the identification of modifiable risk factors in this vulnerable group.

Despite these mechanistic uncertainties, our study provides novel real-world epidemiological evidence from a large clinical population, addressing a critical knowledge gap. While preclinical data cannot be directly extrapolated to clinical outcomes, our findings generate important hypotheses that warrant prospective investigation through well-designed clinical studies and mechanistic research to clarify the relationship between cannabis use and pancreatic cancer risk in patients with chronic pancreatitis.

Our study’s strengths include the large sample size, propensity score matching to minimize confounding bias, and implementation of sensitivity analysis. The differential follow-up time between cohorts warrants consideration, with the shorter follow-up time in the CUD cohort being a potential factor for lower PC case detection. However, Cox proportional hazards regression inherently accounts for differential follow-up by estimating the instantaneous hazard at any given time point among those still at risk, rather than relying on cumulative event counts [18]. However, the study is limited by its observational nature, which means that only associations, not causal relationships, can be inferred. TriNetX relies on coded diagnostic data from electronic health records, which may be subject to coding inaccuracies and incomplete capture of clinical information. Moreover, the lack of detailed information on the frequency, duration, or type of cannabis use restricts the ability to determine dose-response relationships. Cannabinoid pharmacokinetics are influenced by numerous variables, and product labeling is often inaccurate, making the quantification of exposure particularly challenging [19–21]. Future studies incorporating more granular data on healthcare utilization patterns, socioeconomic variables, and provider-level factors would be valuable in clarifying the influence of potential unmeasured confounders on our findings.

In conclusion, these exploratory findings suggest an association between CUD and reduced PC detection during available follow-up in patients with CP. CUD was also associated with a modestly increased risk of AP flares, which aligns with emerging evidence linking cannabis use to acute pancreatitis episodes. Given the limitations of our retrospective observational study, these associations require validation in prospective studies with longer, standardized follow-up and comprehensive ascertainment of cancer outcomes before clinical implications can be determined. Future research should also investigate potential mechanisms and determine whether the observed differences reflect true biological effects or detection bias.

## Data Availability

Data was accessed using TrinetX Database. More information is available at https://trinetx.com/.
